# Artificial Intelligence-Assisted Lung Perfusion Quantification from Spectral CT Iodine Map in Pulmonary Embolism

**DOI:** 10.3390/diagnostics15151963

**Published:** 2025-08-05

**Authors:** Reza Piri, Parisa Seyedhosseini, Samir Jawad, Emilie Sonne-Holm, Camilla Stedstrup Mosgaard, Ekim Seven, Kristian Eskesen, Ole Peter Kristiansen, Søren Fanø, Mathias Greve Lindholm, Lia E. Bang, Jørn Carlsen, Anna Kalhauge, Lars Lönn, Jesper Kjærgaard, Peter Sommer Ulriksen

**Affiliations:** 1 Department of Nuclear Medicine, Odense University Hospital, 5000 Odense, Denmark; 2 Department of Clinical Research, University of Southern Denmark, 5230 Odense, Denmark; 3 Department of Radiology, Copenhagen University Hospital—Rigshospitalet, 2100 Copenhagen, Denmark; 4 Department of Cardiology, Copenhagen University Hospital—Rigshospitalet, 2100 Copenhagen, Denmark; 5 Department of Cardiology, Copenhagen University Hospital—Amager and Hvidovre, 2650 Hvidovre, Denmark; 6 Department of Clinical Medicine, University of Copenhagen, 2100 Copenhagen, Denmark; 7 Department of Cardiology, Copenhagen University Hospital—Gentofte, 2900 Hellerup, Denmark; 8 Department of Cardiology, Copenhagen University Hospital—Bispebjerg & Frederiksberg Hospital, University of Copenhagen, 2000 Copenhagen, Denmark; 9 Department of Cardiology, Copenhagen University Hospital—Herlev, 2730 Herlev, Denmark; 10 Department of Cardiology, Zealand University Hospital—Roskilde, 4000 Roskilde, Denmark; 11 Faculty of Health and Medical Sciences, University of Copenhagen, 2100 Copenhagen, Denmark

**Keywords:** pulmonary embolism, dual-energy computed tomography, perfusion defect, artificial intelligence, Miller score, iodine map, DECT, Spectral CT

## Abstract

**Introduction**: This study evaluated the performance of automated dual-energy computed tomography (DECT)-based quantification of perfusion defects (PDs) in acute pulmonary embolism and examined its correlation with clinical parameters. **Methods**: We retrospectively analyzed data from 171 patients treated for moderate-to-severe acute pulmonary embolism, who underwent DECT imaging at two separate time points. PDs were quantified using a fully automated AI-based segmentation method that relied exclusively on iodine perfusion maps. This was compared with a semi-automatic clinician-guided segmentation, where radiologists manually adjusted thresholds to eliminate artifacts. Clinical variables including the Miller obstruction score, right-to-left ventricular diameter ratio, oxygen saturation, and patient-reported symptoms were also collected. **Results**: The semiautomatic method demonstrated stronger correlations with embolic burden (Miller score; r = 0.4, *p* < 0.001 at follow-up) and a negative correlation with oxygen saturation (r = −0.2, *p* = 0.04). In contrast, the fully automated AI-based quantification consistently produced lower PD values and demonstrated weaker associations with clinical parameters. **Conclusions**: Semiautomatic quantification of PDs currently provides superior accuracy and clinical relevance for evaluating lung PDs in acute pulmonary embolism. Future multimodal AI models that incorporate both anatomical and clinical data may further enhance diagnostic precision.

## 1. Introduction

Pulmonary embolism (PE) is a leading cause of cardiovascular morbidity and mortality, with an annual incidence of 60–70 cases per 100,000 population [1]. Traditional diagnostic tools like computed tomography angiography (CTA) provide anatomical details and have an essential role in the evaluation of PE, but often miss functional impacts, such as perfusion defects (PDs) [2,3]. Dual-energy computed tomography (DECT) has emerged as a promising solution, offering both anatomical and functional information [4,5]. DECT visualizes and quantifies pulmonary PDs through the generation of iodine maps that reflect pulmonary perfusion, facilitating the detection of PDs associated with PE [6,7]

Despite these advancements, current diagnostic practices often fall short in evaluating the functional severity of PE. CTA alone does not offer insight into the extent of hemodynamic compromise or the perfusion status of lung parenchyma. This limitation can hinder risk stratification and prognostication. DECT iodine perfusion imaging addresses this gap by enabling visualization of perfusion abnormalities in real-time. However, quantifying these abnormalities in a standardized and clinically meaningful way remains a challenge.

The integration of artificial intelligence (AI) into medical imaging has further revolutionized the evaluation of PE. AI-driven algorithms can automatically quantify PDs from DECT data, enhancing diagnostic accuracy and efficiency [8]. Studies have demonstrated that AI-based assessments correlate well with established clinical parameters, such as right-to-left ventricular (RV/LV) diameter ratio and the Miller score, which are critical in evaluating the severity of PE and predicting patient outcomes [9]. Despite the promising advancements in automated quantification, semi-automatic methods continue to play a crucial role [10]. These approaches allow for manual adjustments based on clinical judgment, potentially capturing nuances that automated systems might overlook. Comparative analyses between AI-based and semiautomatic methods are essential to validate the reliability and clinical applicability of automated quantification tools. To our knowledge, no prior studies have directly compared a commercially available AI-based automatic segmentation method with a semi-automatic approach using DECT iodine maps in patients with PE. This lack of head-to-head validation leaves a gap in understanding how well-automated tools reflect true perfusion deficits and their clinical consequences.

In addition, correlating clinical parameters—such as total Miller score [9], tricuspid regurgitant gradient [9,11], oxygen requirements, oxygen saturation, respiratory rate, dyspnea [11,12], and six-minute walk test [9,12]—with the quantified PDs may offer valuable insight into the clinical relevance of these imaging findings. These correlations help assess how accurately the calculated PDs reflect the physiological and functional impact of pulmonary embolism, supporting their potential use in risk stratification and clinical decision-making. In this study, the primary objective is to compare automated AI-based quantification of pulmonary PDs using DECT with semi-automatic, clinician-guided quantification in patients with acute pulmonary embolism (PE). The secondary objective is to evaluate the associations between perfusion defect measurements and various clinical parameters at baseline and follow-up, aiming to enhance diagnostic accuracy and prognostic assessment in the management of acute PE. Ultimately, this study contributes novel data on the relative strengths and limitations of automatic vs. semi-automatic segmentation, offering practical insights into how automated tools can be integrated into clinical workflows.

## 2. Materials and Methods

### 2.1. Study Design

This retrospective single-center study (approval from the institutional review board, Ref. No. 20036558) was conducted between August 2019 and January 2021 [13]; a total of 201 patients treated for moderate-to-severe acute PE underwent DECT. We included only patients with intermediate–high risk pulmonary embolism, defined as hemodynamically stable individuals showing evidence of right ventricular dysfunction both biochemically (elevated biomarkers) and on imaging. These patients were scanned 48–96 h after PE diagnosis and after three months. Factors for exclusion were imaging artifacts, inadequate vascular contrast, pulmonary metastases, infections, and pleural effusions.

### 2.2. CT Acquisition

CT scans were performed using a Revolution CT system (GE Healthcare, Milwaukee, WI, USA), which is a single-source dual-energy scanner. The imaging protocol involved fast-switching between tube voltages of 80 and 140 kV, with data captured during a single breath-hold. Scanning was conducted in a caudo-cranial direction, extending from just below the diaphragm to the apex of the lungs. For the pulmonary CTA sequence, images were reconstructed using a virtual monoenergetic level of 62 keV. A split-bolus technique was applied for contrast delivery, utilizing a power injector connected to an antecubital vein. The initial bolus consisted of 35 mL of Omnipaque 350 mg I/mL (GE Healthcare, Milwaukee, WI, USA), followed by a 15 mL saline flush. This was succeeded by a second bolus of 15 mL Omnipaque 350 mg I/mL and a subsequent 30 mL saline flush. The injection rate was maintained at 5.0 mL/s. Image acquisition triggered 4.6 s after contrast enhancement in the pulmonary trunk reached 120 Hounsfield Units, as determined by bolus tracking. Key scanner parameters included an 80 mm detector collimation, 0.5 s gantry rotation time, pitch of 1.531, a 500 mm scan field of view, and a display field of view tailored to the individual patient. Images were reconstructed with a slice thickness and reconstruction increment of 0.625 mm.

### 2.3. CTA Interpretation and Obstruction Score

All CTA scans were independently reviewed by a radiologist (SJ) using a PACS workstation (Impax v6.7.0.6011, AGFA Healthcare, Mortsel, Belgium). Obstruction severity on CTA was quantified using the refined modified Miller score, ranging from 0 to 40 points [14]. The reviewer was blinded to prior CTA interpretations, as well as to iodine perfusion maps, PD, and RV/LV diameter ratios. The diagnosis of acute PE was based on the identification of intraluminal filling defects in pulmonary arteries in clinically suspected patients. The location, extent, and degree of obstruction caused by emboli were documented. The same radiologist assessed the RV/LV diameter ratio using an axial view method [15] two weeks later, with the scans presented in randomized order.

### 2.4. Quantification of Total Lung Volume and PD

Automated calculations of total lung volume and PDs were performed using a commercially available dual-energy post-processing software (Thoracic VCAR, GSI Pulmonary Perfusion, GE Healthcare, AW Server v3.2 Ext 2.0, Milwaukee, WI, USA) on a GE AW workstation. The Thoracic VCAR application provides visualization of pulmonary perfusion by applying a color-coded iodine overlay on lung parenchyma, highlighting regions with relatively reduced iodine concentration indicative of perfusion deficits. An algorithm-driven automatic threshold is used to define low-iodine-concentration regions within the lung, which can be adjusted manually; however, no manual adjustments were made during the automatic analysis. Automatic quantification yields total lung volume in cubic centimeters and PDs as a percentage of total lung volume.

This verified proprietary software generates perfusion overlays using internal algorithms based on iodine density, but the exact segmentation methodology is not publicly disclosed due to its commercial nature. For the semiautomatic analysis, three radiologists (RP, PSH, and PSU) independently reviewed the iodine perfusion maps and adjusted thresholds manually to optimize the delineation of perfusion defects. The manual criteria involved including wedge-shaped, peripheral areas with reduced iodine concentration consistent with embolic patterns, while excluding regions suggestive of artifacts. These excluded areas comprised non-wedge-shaped regions, areas adjacent to pleura or fissures, motion-related or beam-hardening artifacts, and perfusion voids lacking anatomical correlates on the corresponding CTA images. This approach was intended to enhance specificity by minimizing the inclusion of artifactual findings as PD.

**Figure 1:**
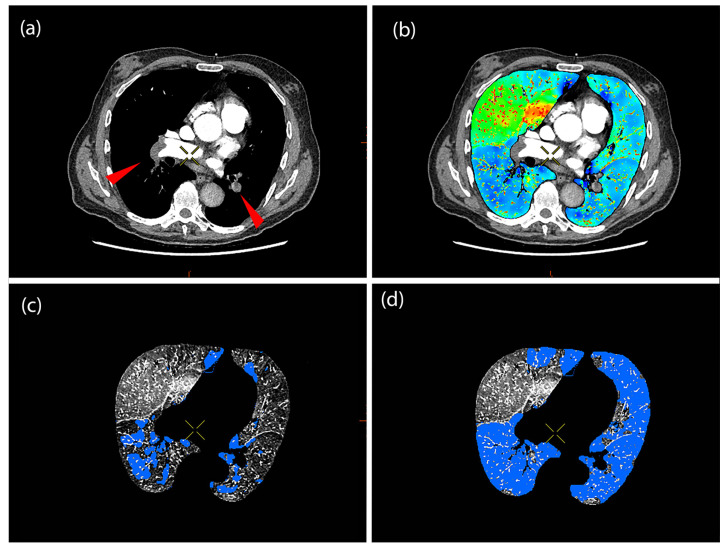
Representative example of perfusion defect assessment in a patient with bilateral basal pulmonary embolism. (**a**) Axial CTA image showing emboli in bilateral lower lobe pulmonary arteries (red arrowheads). (**b**) Corresponding iodine perfusion map from DECT, demonstrating reduced perfusion in both lower lobes and partially in the upper lobes. (**c**) Automated segmentation using Thoracic VCAR software, with perfusion defects shown in blue; note under-segmentation relative to the extent of visible perfusion abnormalities. (**d**) Semi-automated segmentation after manual threshold adjustment, showing more extensive perfusion defects involving bilateral lower lobes and anterior regions of the upper lobes, aligning more closely with the embolic distribution seen on CTA.

An illustrative example is shown in Figure 1, where the automated segmentation underestimates the extent of perfusion defects, particularly in the lower lobes, whereas the semi-automated method corrects this by incorporating perfusion defects in regions corresponding to the embolic distribution seen on CTA.

### 2.5. Statistical Methods

All statistical analyses were performed using Python 3.12.3 (Spyder IDE 6.0.7) with pandas, scipy, statsmodels, and seaborn. Continuous variables were reported as means ± standard deviations for evenly distributed values and medians (P25–P75) for skewed data, and categorical variables were presented as counts and percentages. A paired *t*-test was used to compare automatically and semi-automatically calculated PDs at baseline and follow-up. Histograms were used to visualize the distributions of PD values across time points. Agreement between the two methods was further analyzed using Bland–Altman plots, which displayed the mean difference (bias) and limits of agreement (±1.96 SD). Interrater reliability was assessed using the intraclass correlation coefficient (ICC) with a two-way random model and consistency agreement, focusing on average measures. The ICC was calculated for all time points, baseline, and follow-up, and statistical significance was determined using an F-test (*p* < 0.05). Differences in ratings between raters were assessed using a one-way analysis of variance, followed by Tukey’s post hoc test for pairwise comparisons (*p* < 0.05). Pearson correlation coefficients were calculated to assess relationships among imaging and clinical variables at 48 h and 3 months. A correlation matrix was generated, and only significant correlations (*p* < 0.05) were reported. Distributions of rater scores at baseline and follow-up were illustrated using violin plots, where the width represents score density and inner quartile lines indicate variability. Histograms compared automatically and semi-automatically calculated PDs across time points. A heatmap was created to visualize significant correlations between clinical and imaging variables. All values are presented with a maximum of one digit after the decimal point, except for *p*-values, which are reported with three digits.

## 3. Results

Table 1 summarizes the descriptive statistics of the patients, including demographic data, PD measurements, and clinical variables at baseline and follow-up. Of the initial 201 patients, scans from 171 patients were suitable for analysis. The mean age was 69.8 years (±13.9), with a near-equal distribution of females (48.1%) and males (51.9%).

### 3.1. Automatic vs. Semiautomatic Method

**Figure 2:**
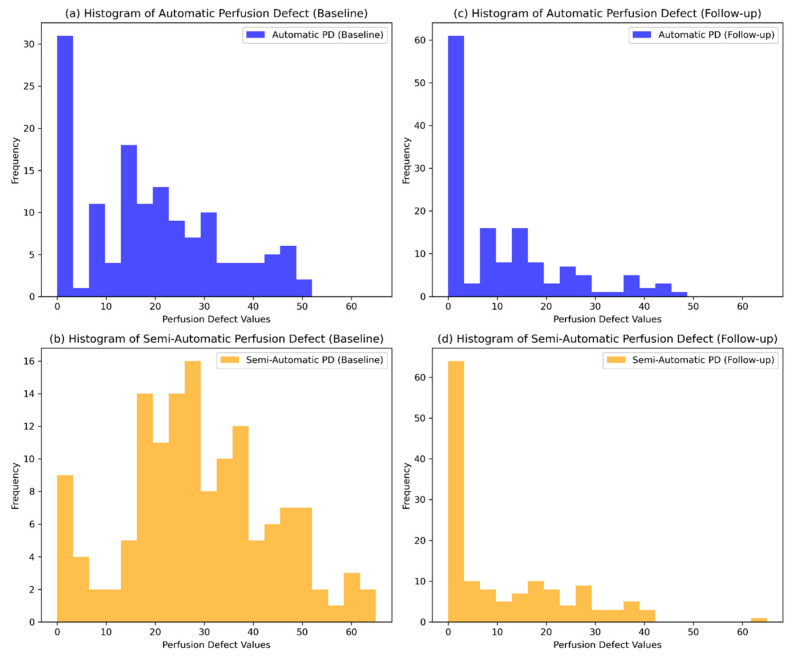
Histograms comparing automatically (blue) and semi-automatically calculated PD values (orange) at baseline and follow-up. (**a**) Histogram of automatically calculated PDs at baseline. (**b**) Histogram of semi-automatically calculated PDs at baseline. (**c**) Histogram of automatically calculated PDs at follow-up. (**d**) Histogram of semi-automatically calculated PDs at follow-up. The *X*-axis represents the PD values, while the *Y*-axis represents the frequency of observations.

Automatically calculated PD values at baseline were significantly lower than those obtained through the semi-automatic method (*p* < 0.001), as shown in Figure 1 and Table 1. At follow-up, the values converged with no significant difference (*p* = 0.89). This indicates a potential underestimation by the fully automatic method in acute-phase scans. Figure 2 presents the histograms of automatically and semi-automatically calculated PDs at baseline and follow-up, illustrating their distributions across time points. To enhance transparency and provide a comprehensive overview of individual patient data, we have included Supplementary Table S2, which lists all automatically and semi-automatically calculated perfusion defect values at baseline and follow-up for each patient.

**Figure 3:**
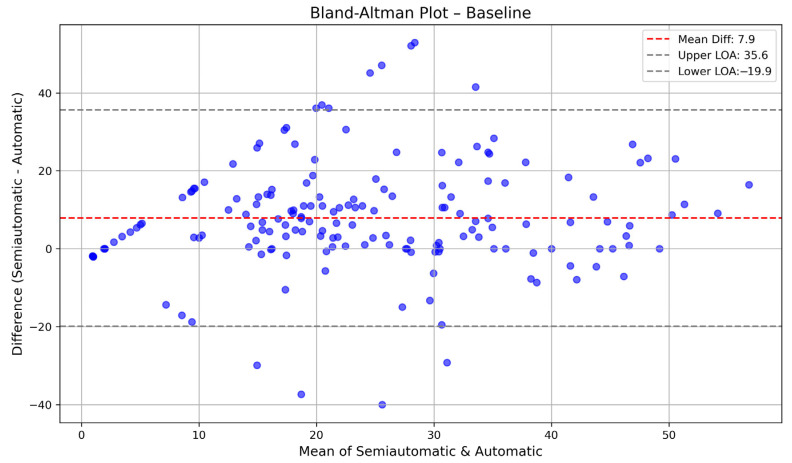
Bland–Altman plot comparing automatically calculated PDs and semi-automatically calculated PDs at baseline. The *X*-axis represents the mean of the two measurements, while the *Y*-axis represents their difference (semi-automatic minus automatic). The red dashed line indicates the mean difference (bias), and the blue dashed lines represent the limits of agreement (mean difference ± 1.96 standard deviations).

**Figure 4:**
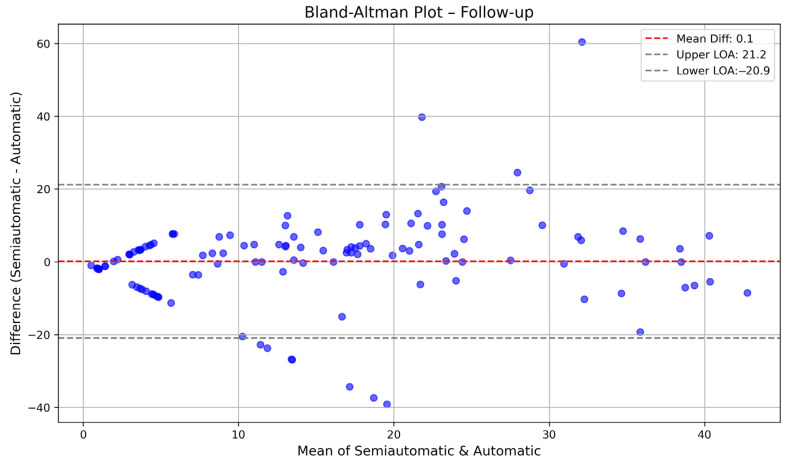
Bland–Altman plot comparing automatically calculated PDs and semi-automatically calculated PDs at follow-up. The *X*-axis represents the mean of the two measurements, while the *Y*-axis represents their difference (semi-automatic minus automatic). The red dashed line indicates the mean difference (bias), and the blue dashed lines represent the limits of agreement (mean difference ± 1.96 standard deviations).

Bland–Altman plots were used to evaluate the agreement between automatically and semi-automatically calculated PDs at baseline, follow-up, and combined time points. At baseline, the mean difference was 7.9, with limits of agreement ranging from −19.9 to 35.6, indicating that the automatic method generally overestimated PDs compared to the semi-automatic method. At follow-up, the mean difference was 0.1, with narrower limits of agreement (−20.9 to 21.2), suggesting better consistency between methods over time. When considering both time points together, the mean difference was 4, with limits of agreement spanning −21.8 to 29.8, reflecting an overall moderate level of agreement. These results indicate that while the two methods showed some discrepancies at baseline, their agreement improved at follow-up. The Bland–Altman plots for baseline are shown in Figure 3 and Figure 4.

### 3.2. Interrater Variability

**Figure 5:**
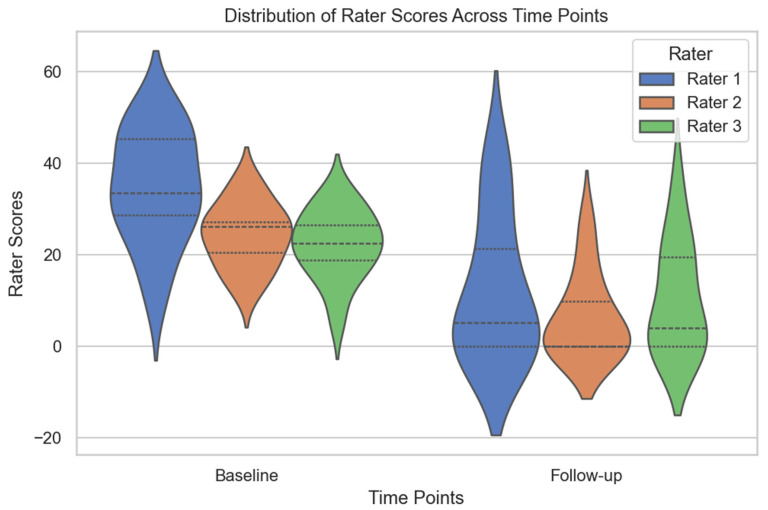
Distribution of rater scores at baseline and follow-up. The violin plot shows the distribution of scores assigned by Rater 1, Rater 2, and Rater 3 at baseline (n = 13) and follow-up (n = 11). The width of each violin reflects the density of scores, with inner lines indicating the interquartile range and the central dot representing the median. The plot illustrates variability in ratings across time points, with no significant differences between raters.

Interrater reliability was assessed using the intraclass ICC with a two-way random model and consistency agreement, focusing on average measures. At baseline and follow-up (n = 24), the ICC for average measures was 0.9 (*p* < 0.001), indicating excellent agreement among the raters. At baseline (n = 13), the ICC for average measures was 0.73 (*p* < 0.001), suggesting good reliability. Similarly, at follow-up (n = 11), the ICC for average measures was 0.8 (*p* < 0.001), reflecting strong consistency in ratings. These results indicate strong inter-rater agreement at both time points, with higher reliability at follow-up. Rater score distributions at baseline and follow-up are shown in Figure 5.

To assess differences in scoring between raters, a one-way ANOVA was conducted, which did not reveal statistically significant differences in ratings (*p* > 0.05). Post hoc analysis using Tukey’s HSD test further confirmed that none of the pairwise comparisons between raters were statistically significant (*p* > 0.05), indicating that no single rater systematically differed from the others in their assessments.

### 3.3. Correlations Among Imaging Variables

At 48 h post PE, the semiautomatic PD showed a strong correlation with the automatic PD (r = 0.5, *p* < 0.001), confirming consistency between the two quantification methods. It also correlated positively with the total Miller score (r = 0.3, *p* = 0.014), reinforcing its association with clot burden. A significant negative correlation was observed with oxygen saturation (r = −0.2, *p* = 0.04), indicating that larger PD were associated with lower oxygen levels.

**Figure 6:**
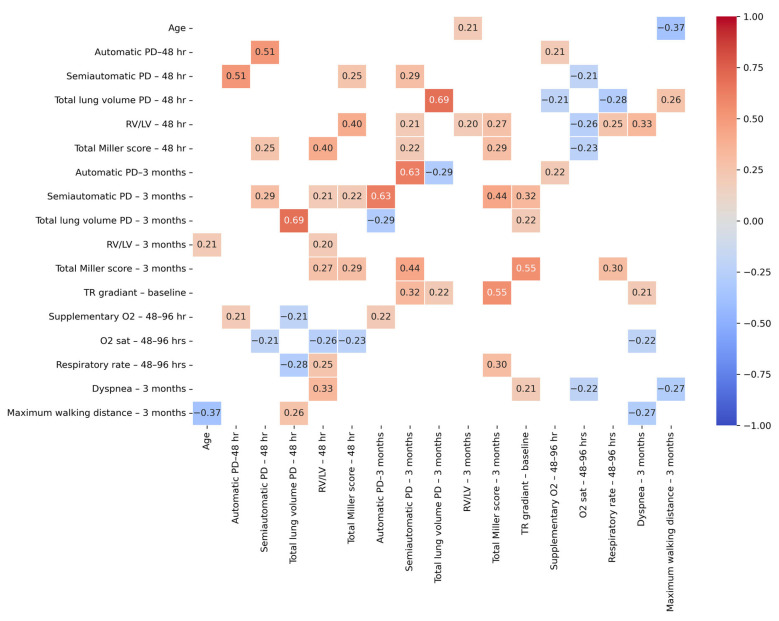
Correlation heatmap displaying only significant correlations (*p* < 0.05) between selected clinical and imaging variables. Non-significant correlations are masked in white. The strength and direction of correlations are represented by the color scale, with red indicating positive correlations and blue indicating negative correlations. The full correlation matrix is provided in the Supplementary Materials. LV: left ventricle; PD: perfusion defect; RV: right ventricle; TR: tricuspid.

At the three-month follow-up, the semiautomatic PD remained significantly associated with its 48 h counterpart (r = 0.3, *p* = 0.005), suggesting temporal stability in perfusion abnormalities. It also showed moderate correlations with the total Miller score (r = 0.4, *p* < 0.001) and a strong correlation with the automatic PD (r = 0.6, *p* < 0.001), supporting the consistency of these imaging markers. A borderline correlation with baseline TR gradient (r = 0.3, *p* = 0.055) suggests a potential association between early right heart strain and persistent PDs. These relationships are visually summarized in Figure 6, which presents a correlation heatmap of significant associations (*p* < 0.05) between selected clinical and imaging variables, while non-significant correlations are masked. The complete correlation matrix is available in Table S2. Overall, the semi-automatic method demonstrated stronger correlations with key clinical parameters—including the Miller score (r = 0.4, *p* < 0.001) and oxygen saturation (r = –0.2, *p* = 0.04)—compared to the fully automatic method. These findings suggest that manual threshold adjustment improves the clinical relevance of perfusion quantification, particularly in the acute phase following PE.

## 4. Discussion

In this study comparing AI-based and radiologist-guided PD quantification of PDs in acute PE, semi-automated measurements demonstrated a stronger correlation with embolic burden (Miller score) and an inverse correlation with oxygen saturation, indicating functional relevance. The automated method, which relied solely on iodine perfusion maps, consistently yielded systematically lower PD values and showed weaker associations with clinical parameters. Currently, no universally accepted gold standard exists for estimating the extent of lung PDs in DECT. In this context, the semiautomatic method employed in this study offers substantial accuracy, benefiting from expert oversight and anatomical insights. Prior research supports our findings, showing that semiautomatic PD volumes correlate moderately with angiographic obstruction scores, whereas fully automated approaches generally have weaker correlations [7,16]. These observations underscore the importance of expert supervision in clinical evaluations of PE.

The principal limitation of our AI model is its reliance exclusively on iodine perfusion maps without incorporating vascular anatomical information from CTA. Previous studies have identified discrepancies between PDs and embolic occlusions, emphasizing the necessity of multimodal imaging integration for accurate interpretation [17,18]. Incorporating CTA information into AI analyses could significantly enhance diagnostic precision. Indeed, recent multimodal AI models integrating perfusion maps, angiographic imaging, and clinical data have shown promise in improving diagnostic accuracy and clinical prognostication [19].

Interpretation of iodine perfusion maps presents inherent challenges due to imaging artifacts such as atelectasis and pleural effusions. Although radiologists actively exclude such artifacts during PD assessments, distinguishing between true PDs and artifacts remains a challenge because of similar attenuation characteristics on iodine maps. Additional technical artifacts, including motion and beam hardening, further complicate interpretation, as previously described in the DECT literature [20,21]. Automated systems, lacking contextual understanding, are especially susceptible to these artifacts, highlighting the interpretive complexity involved in perfusion imaging.

When evaluating the semiautomatic approach, we also assessed interrater variability. The ICC for perfusion defect measurements among radiologists indicated strong agreement, which was somewhat superior to what was reported earlier [22,23], probably because it was between three raters. This level of consistency among human readers highlights that the observed discrepancy between AI and semiautomatic results is unlikely to be explained by subjective variation among radiologists. In fact, the difference between AI and semi-automatic methods exceeded the differences observed between individual radiologists, suggesting that the AI’s limited input and interpretive scope played a more substantial role than human variability in explaining the disagreement in PD quantification.

While the semi-automatic method was used as a reference in our comparison, we acknowledge that this does not represent an absolute gold standard. The accuracy of semi-automatic segmentation is inherently dependent on radiologist input and may be influenced by subjective interpretation. However, in this study, interrater reliability was assessed and found to be high (ICC = 0.9 across all time points), supporting the consistency of the segmentation results across observers. We have therefore used the semi-automatic method as a practical reference standard, but we recognize that even expert-guided segmentation is not immune to variability, which should be considered when interpreting the results.

Our study has some limitations. The relatively small sample size and single-center design limit generalizability. The absence of a control group without PE restricts the evaluation of diagnostic accuracy. Moreover, scanner vendor incompatibility and post-processing software constraints resulted in inconsistent follow-up imaging, introducing potential selection bias. Additionally, although multiple radiologists participated, inherent subjectivity and non-blinded assessment of embolic locations may have influenced outcomes. Pulmonary perfusion maps derived from DECT are based on iodine distribution at the exact moment of imaging. Consequently, various technical parameters, such as contrast volume, iodine concentration, injection flow rate, and scan timing, can influence the final images. As a result, the observed relative perfusion deficits might not accurately represent actual biological perfusion abnormalities.

It is important to note that perfusion defects, as visualized on DECT iodine maps, primarily reflect the embolic burden within the pulmonary vasculature. However, clinical parameters such as oxygen saturation and even right-sided pressure indicators like tricuspid regurgitant gradient are influenced by multiple factors beyond embolic obstruction. Co-morbidities including underlying cardiac dysfunction, pre-existing pulmonary disease, and overall cardiopulmonary reserve may all impact clinical presentation and outcomes independently of the perfusion defect extent. This may partly explain the limited correlation observed between fully automatic PD quantification and certain clinical markers.

Future research should prioritize developing multimodal AI models that integrate DECT perfusion maps with angiographic and clinical data, enhancing diagnostic precision. Larger, multicenter datasets will be necessary for validating these advanced methods. Prospective studies should explore the predictive capabilities of DECT-based PD quantification for clinical outcomes, including exercise tolerance, chronic thromboembolic pulmonary hypertension, and mortality. Standardizing PD quantification across platforms and vendors is crucial for reproducibility and widespread clinical adoption. Additionally, investigating AI-enhanced DECT as a diagnostic tool in emergency settings warrants further research. To improve diagnostic performance, future multimodal AI models should aim to integrate multiple data sources beyond iodine maps alone. These may include anatomical information from CTA, patient-level clinical variables such as right-to-left ventricular diameter ratio, cardiac biomarkers, oxygenation status, and possibly echocardiographic data. Deep learning models that combine imaging and structured clinical data—such as hybrid convolutional neural networks with attention mechanisms or transformer-based architectures—may be well-suited to capture complex interactions and improve outcome prediction. Training such models on large, multicenter datasets with robust clinical endpoints could enhance generalizability and clinical impact.

## 5. Conclusions

Semiautomatic quantification of perfusion defects in DECT remains the most reliable method for assessing perfusion defects in acute pulmonary embolism. It demonstrates strong correlations with embolic burden and clinical markers of impairment. These findings support the continued involvement of clinicians in current practice and encourage the further development of multimodal AI methods. Future studies with larger datasets and integrated imaging modalities are essential to fully harness the potential of AI in clinical pulmonary embolism management.

## Figures and Tables

**Table 1:** Descriptive statistics of the included patients (*n* = 201) *.

Age	69.8 ± 13.9
Sex (%)	97 female (48.1), 104 male (51.9)
Automatic PD (%)—48 h (*n* = 171)	20.1 ± 14
Semiautomatisk PD (%)—48 h (*n* = 171)	28 ± 15.4
Total lung volume (mm^3^)—48 h (*n* = 171)	3058.7 ± 980
RV/LV—48 h	1.34 ± 3.1
Total Miller score—48 h	14.3 ± 4.9
Automatic PD (%)—3 months (*n* = 167)	11.8 ± 11.7
Semiautomatisk PD (%)—3 months (*n* = 167)	11.94± 13.7
Total lung volume (mm^3^)—3 months (*n* = 167)	3123.7 ± 921
RV/LV—3 months	1.2± 3.3
Total Miller score—3 months	1.1± 3.3
TR-gradient (mmHg)—baseline	28.1 ± 6.8
Supplementary oxygen (liter)—48–96 h	1 ± 7.3
Oxygen saturation (%)—48–96 h	96.7 ± 1.9
Respiratory rate (per minute)—48–96 h	17.3 ± 2.8
Dyspnea **—3 months	1.5 ± 3.5
Six-minute walk test (meter)—3 months	468.8 ± 126.5

* Descriptive statistics are expressed as mean ± standard deviation. ** The dyspnea score ranges from 0 (no dyspnea) to 10 (unbearable), based on patient self-assessment. LV: left ventricle; PD: perfusion defect; RV: right ventricle; TR: tricuspid.

## Data Availability

The data presented in this study are available on request.

## Supplementary Materials

The following supporting information can be downloaded at: https://www.mdpi.com/article/10.3390/diagnostics15151963/s1, Table S1: Automatically and semi-automatically calculated perfusion defect values at baseline and follow-up for each patient; Table S2: Full correlation matrix including all selected clinical and imaging variables.

## Abbreviations

The following abbreviations are used in this manuscript:

AI	Artificial Intelligence
CTA	Computed Tomography Angiography
DECT	Dual-Energy Computed Tomography
ICC	Intraclass Correlation Coefficient
LV	Left Ventricle
PD	Perfusion Defect
PE	Pulmonary Embolism
RV	Right Ventricle
RV/LV	Right-to-Left Ventricular Diameter Ratio
TR	Tricuspid
VCAR	Volume Computer-Assisted Reading (Thoracic VCAR)
